# Agreement between clinical and non-clinical digital manometer for assessing maximal respiratory pressures in healthy subjects

**DOI:** 10.1371/journal.pone.0224357

**Published:** 2019-10-24

**Authors:** Rodrigo Torres-Castro, Nicolás Sepúlveda-Cáceres, Rodrigo Garrido-Baquedano, Marisol Barros-Poblete, Matías Otto-Yáñez, Luis Vasconcello, Roberto Vera-Uribe, Homero Puppo, Guilherme Fregonezi

**Affiliations:** 1 Departmento de Kinesiología, Facultad de Medicina, Universidad de Chile, Santiago de Chile, Chile; 2 Programa Nacional de Ventilación Mecánica No Invasiva, Ministerio de Salud, Santiago de Chile, Chile; 3 Escuela de Kinesiología, Universidad Autónoma de Chile, Santiago de Chile, Chile; 4 PneumoCardioVascular Lab, Departamento de Fisioterapia & Hospital Universitário Onofre Lopes - Empresa Brasileira de Serviços Hospitalares (EBSERH), Universidade Federal do Rio Grande do Norte (UFRN), Natal, Rio Grande do Norte, Brazil; University of Maiduguri College of Medical Sciences, NIGERIA

## Abstract

Measurement of respiratory muscles strength such as maximal inspiratory pressure (MIP) and maximal expiratory pressure (MEP) are used to detect, diagnose and treat respiratory weakness. However, devices used for these measurements are not widely available and are costly. Currently, the use of a digital manometer is recommended. In industry, several inexpensive devices are available, but these have not been validated for clinical use. Our objective was to determine the agreement between maximal respiratory pressures obtained with a clinical digital manometer and that with a non-clinical digital manometer in healthy volunteers. We assessed the height, weight, lung function, MIP, and MEP of healthy volunteers. To compare pressures obtained by each type of digital manometer, a parallel approach configuration was used. The agreement was measured with the Intraclass Coefficient Correlation (ICC) and the Bland-Altman plot. Twenty-seven participants (14 men) were recruited with a median age of 22 (range: 21–23) years. Each participant underwent three measurements to give a total of 81 measurements. The mean MIPs were 90.8 ± 26.4 (SEM 2.9) and 91.1 ± 26.4 (SEM 2.9) cmH_2_O for the clinical and non-clinical digital manometers, respectively. The mean MEPs were 113.8 ± 40.4 (SEM 4.5) and 114.5 ± 40.5 (SEM 4.5) cmH_2_O for the clinical and non-clinical digital manometers, respectively. We obtained an ICC of 0.998 (IC 0.997–0.999) for MIP and 0.999 (IC 0.998–0.999) for MEP. There is a high agreement in the values obtained for MIP and MEP between clinical and non-clinical digital manometers in healthy volunteers. Further validation at lower pressures and safety profiling among human subjects is needed.

## Introduction

Assessment of respiratory muscle strength is clinically useful for monitoring respiratory muscle weakness [1]. In clinical practice, the measurement of respiratory muscle strength plays an important role in the recognition of respiratory muscles weakness in symptomatic patients. Respiratory muscle weakness has been reported in several conditions including cardiac disease [2], chronic respiratory diseases [3,4], and, most notably, in neuromuscular diseases [5].

Conventionally, measures of maximal static inspiratory pressure (MIP) and maximal expiratory pressure (MEP) from the mouth have been used to investigate respiratory muscle weakness. These measures are noninvasive, well-tolerated, simple to perform, and normal values for adults and children have been reported by several authors in different countries [6,7]. The recommended instruments for measuring maximal respiratory pressures at the mouth are digital manometers [1]. These are portable, easy to use device that enables the assessment of chronic patients at home. They also include friendly software that can help the evaluator to determine acceptable and optimal results.

Validation data for the use of portable digital mouth pressure meters in clinical practice and at the bedside has existed since 1994 [8]. Moreover, the use of digital electronic transducers or digital manometers was recommended in the American Thoracic Society/European Respiratory Society (ATS/ERS) guidelines more than fifteen years ago, while it was considered that aneroid manometers were characterized by low precision and accuracy [1]. Nevertheless, in most low and middle-income countries, electronic transducer or digital manometers are not widely used because the cost is a major limiting factor for the use of digital devices in many regions.

Electronic transducers are not a new technology and they have been widely applied in the industry (such devices can be referred to as non-clinical devices). A wide range of non-clinical devices is available for industrial use. They are cheap and may have potential clinical applicability. However, health regulation agencies in some countries do not allow their use in clinical practice. The purpose of the present study was to determine the degree of agreement between two hand-held digital manometers, a commercial clinically-validated manometer, and a non-clinical manometer.

## Methods

### Study design

We conducted a cross-sectional study, with selection of a convenience sample. During the development of the study, we followed the recommendations of the Guidelines for Reporting Reliability and Agreement Studies (GRRAS) [9].

### Participants

We recruited volunteers who self-reported as healthy and were aged between 18 and 40 years. We excluded participants with spirometric values below predicted values (80% of forced vital capacity, FVC, and forced expiratory volume at the first second, FEV_1_). We also excluded those with chronic respiratory disease (i.e., asthma, chronic obstructive pulmonary disease, or pulmonary fibrosis), acute respiratory disease in the preceding month, or cardiovascular, cerebral, musculoskeletal, or neuromuscular disease. Finally, we also excluded those with a body mass index (BMI) ≥35, an inability to understand instructions, and those who failed to perform the tests.

This study was approved by the ethics committee for research in humans of the Faculty of Medicine at the University of Chile, number 024–2016, and adheres to the Declaration of Helsinki. All volunteers have signed informed consent.

### Physiological measurements

Respiratory muscle strength: The maximal respiratory pressure was assessed with measurements of MIP and MEP (ATS/ERS 2002) [10] with a respiratory pressure meter. The respiratory pressure meter is a hand-held instrument designed for rapid assessment of inspiratory and expiratory muscle strength. The result of each measurement is presented in units of cmH_2_O in a liquid crystal display screen.

The clinical digital manometer used was the MicroRPM^®^ digital manometer (Micro Medical, London, England), which has a range between -300 and +300, a resolution of 1.0 cmH_2_O, and accuracy of ± 3% cmH_2_O. The non-clinical digital manometer used was a PCE-005^®^ digital manometer (PCE Holding GmbH, Hamburg, Germany) calibrated between -351.5 and + 351.5 cmH_2_O with a resolution of 0.1 cmH_2_O and accuracy of ± 0.2%. The PCE-005^®^ digital manometer is a differential pressure manometer used to measure pressure in pneumatic, compressor, and pump installations, as well as valves, tanks, and heating, ventilation, and air conditioning (HVAC) systems. The PCE-005^®^ digital manometer was tested and calibrated by an ISO 9000 certified company. It is both portable and small.

To evaluate the MIP, participants had to exhale the greatest volume of air they could until the residual volume (RV) was reached. This was followed by maximal inspiratory effort, and they held this maximal inspiration for at least 1 second, according to the technique of Black & Hyatt [11] To evaluate the MEP, participants were instructed to perform maximal inspiration up to total lung capacity (TLC), followed by a sustained maximal expiratory effort up to the residual volume, sustaining this maximal expiration for at least 1 second, according to the technique of Black & Hyatt [11].

**Fig 1:**
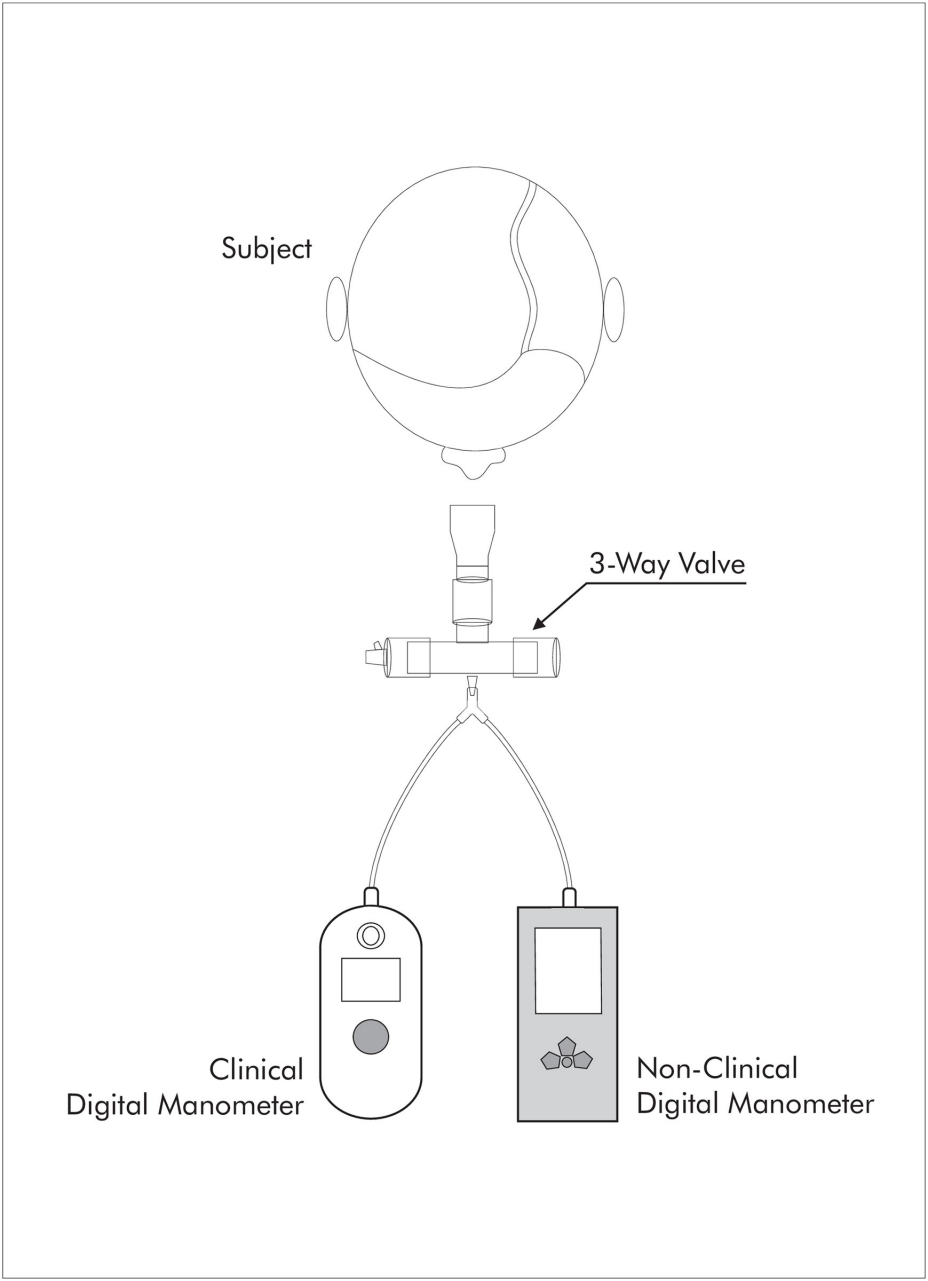
Schematic representation of the model.

**Fig 2:**
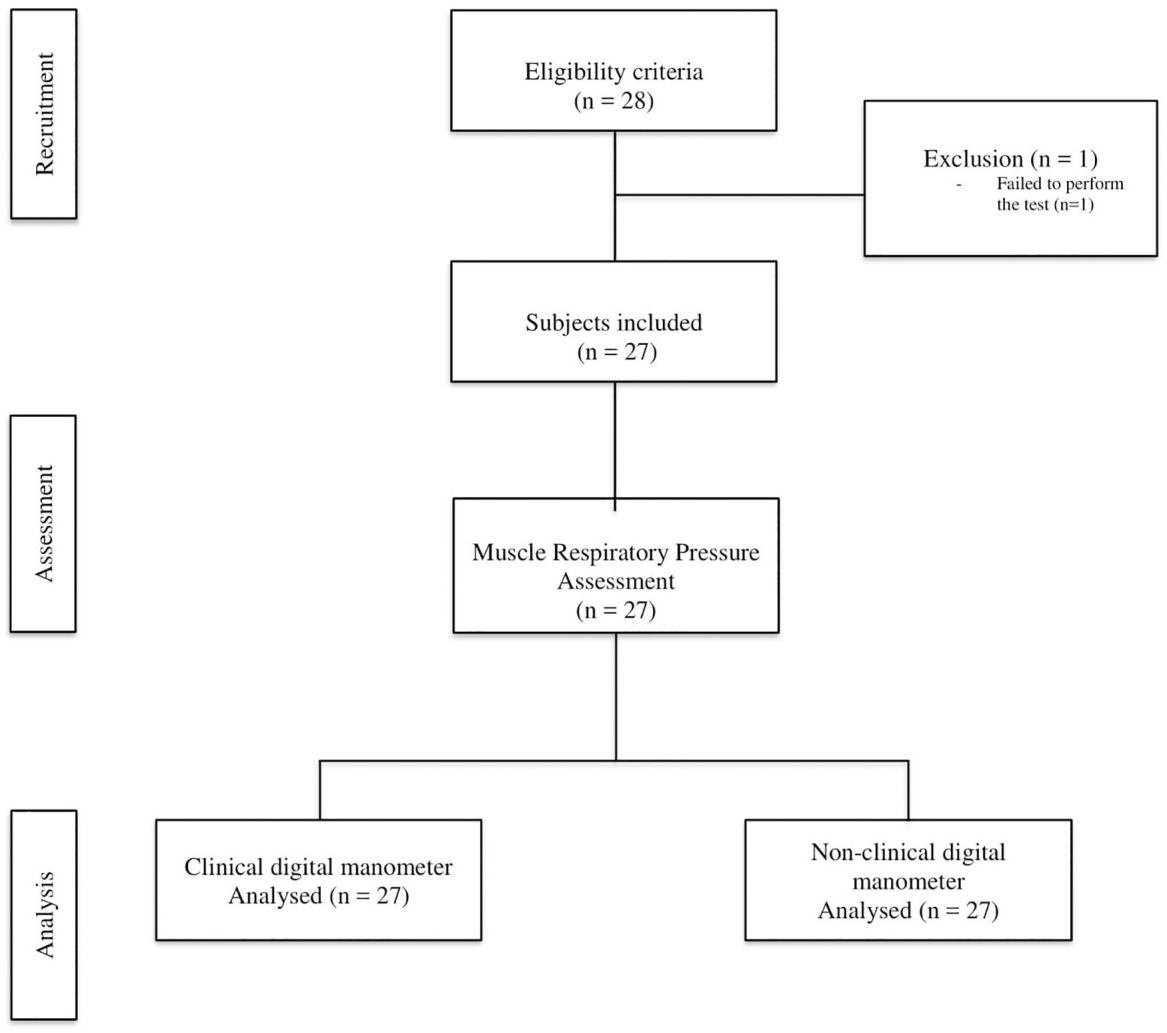
Study flowchart.

All pressures were measured with a nasal clip and a dive mouthpiece connected to a one-way valve (Nif-tee, Hudson, USA) to prevent air leaks. The protocol was applied until three maneuvers that were reproducible were obtained, with a maximum of eight maneuvers. The values could not differ by more than 10% from the highest value, and between each attempt, there was be a rest interval of at least 60 seconds [12]. Each attempt was registered simultaneously by the two pressure manometers, which were connected in parallel (Fig 1). The order of maneuvers (MIP or MEP) was determined with simple randomization (Fig 2).

Pulmonary function: Spirometry was performed with a portable spirometer Microlab NK6 (Micromedical, London, England). We measured the FVC, FEV_1_, FEV_1_/FCV coefficient, and peak expiratory flow (PEF). Results are expressed in absolute values and percentages according to the reference values and all procedures were applied in accordance with the American Thoracic Society guidelines [13].

Anthropometric measurements: We measured the height (cm) and weight (kg) with a SECA 700 stadiometer (SECA GmbH, Hamburg Germany).

### Statistical analysis

The sample size, for agreement studies, required for the results to have the appropriate precision was 77 measurements, for a discordance rate a = 0.05 and a tolerance probability ß = 90% [14]. We verified the normality of data distributions with the Kolmogorov-Smirnov test. Continuous variables are presented as the mean ± standard deviation or as the median and 25th–75th percentiles, as appropriate. To establish data concordance, the similarity between values obtained by each manometer in the evaluation of maximal pressures was evaluated with the Intraclass Correlation Coefficient (ICC). The Bland-Altman method was used to graphically analyze the degree of agreement between measures obtained by the clinical and non-clinical digital manometers [15]. Inferential data analysis was performed with SPSS 23.0 (IBM, Chicago, USA) and GraphPad Prism 6.01 for Windows (GraphPad Software, La Jolla, California).

## Results

We recruited 27 participants (14 male and 13 female), with a median age of 22 (range 21–23) years. The mean (SD) height, weight, and BMI of participants was 167 ± 9.8 cm, 67.5 ± 13.1 kg, and 23.5 ± 3.9 kg/m^2^ respectively. Values obtained for lung function were significantly within the normal range (Table 1).

**Table 1:** Anthropometric and spirometric values of healthy.

Variable	
Male / Female (all)	14 /13 (27)
Age (y)*	22 (21–23)
Height (cm)	167 ± 9,8
Weight (kg)	67,5 ± 13,1
BMI (kg/m2)	23,5 ± 3,9
FVC (L)	4.7 ± 1.1
FVC (%)	111.6 ± 11.4
FEV_1_ (L)	4.0 ± 0.9
FEV_1_ (%)	111.8 ± 11.9
FEV_1_ /FVC	0.85 ± 0.04
PEF (lpm)	517.3 ± 99.2
PEF (%)	110.2 ± 13.9

Values are expressed as the mean ± SD. BMI: Body mass index; FVC: Forced vital capacity; FEV_1_: Forced expiratory volume at the first second; PEF: Peak expiratory flow.

*Data presented as a median (P25-P75)

**Fig 3:**
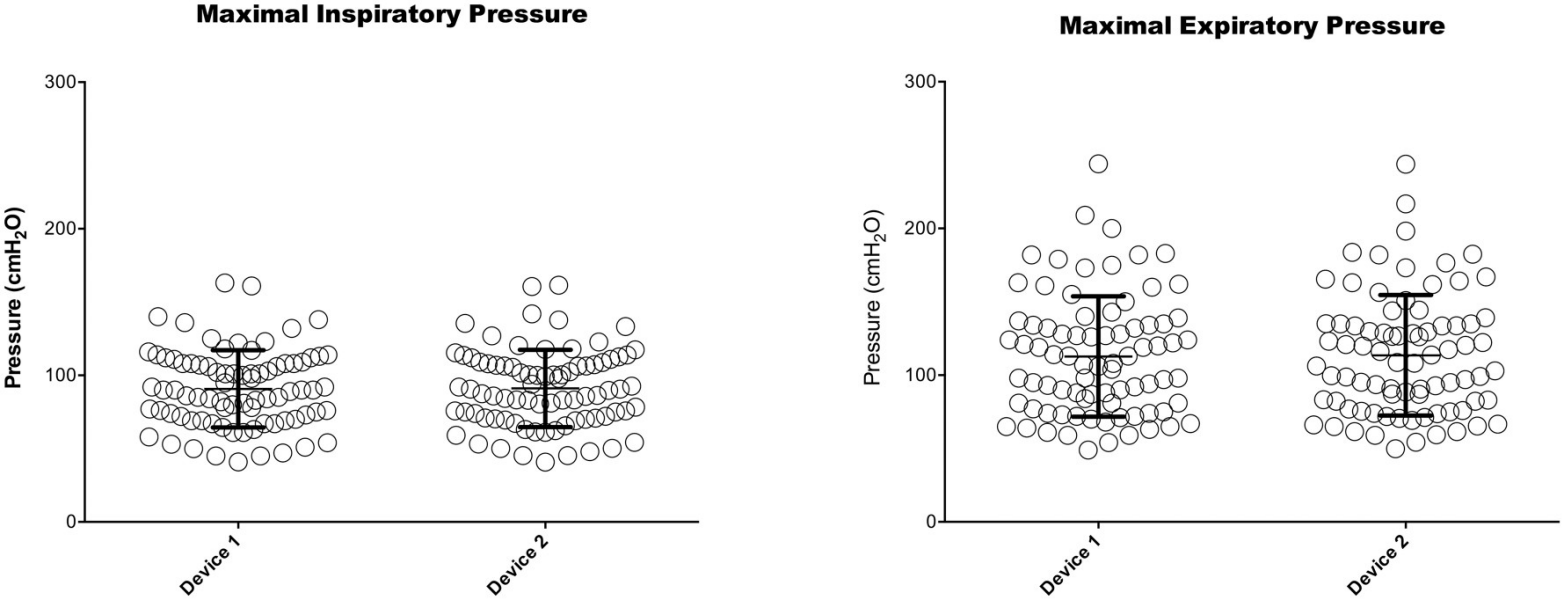
Absolute values of MIP and MEP in device 1 and device 2.

We obtained mean MIPs of 90.8 ± 26.4 (SEM 2.9) cmH_2_O and 91.1 ± 26.4 (SEM 2.9) cmH_2_O for the clinical and non-clinical digital manometers, respectively. The mean MEPs were 113.8 ± 40.4 (SEM 4.5) cmH_2_O and 114.5 ± 40.5 (SEM 4.5) cmH_2_O for the clinical and non-clinical digital manometers, respectively (Fig 3 and Table 2). In women, values of 70.6 ± 15.6 cmH_2_O (SEM 2.6) and 82 ± 19.9 (SEM 3.3) cmH_2_O were obtained for MIP and MEP, respectively. In men, values of 109.2 ± 19.7 (SEM 3.1) cmH_2_O and 140.9 ± 34.7 (SEM 5.4) cmH_2_O were obtained for MIP and MEP, respectively (S1 Table). These MIP values are within normal reference values from both the ATS and Black & Hyatt recommendations. However, the MEP values are 10% below the reference values given by Black & Hyatt and 3 of the six reference values recommended by the ATS [1].

**Table 2:** Values of maximal respiratory pressure of both manometers.

	Device	Mean ± SD (range)	ICC (95% CI)
Maximal inspiratory pressure (cmH_2_O)	Clinical digital manometer	90.8 ± 26.4 (41–163)	0.998 (0.997–0.999)
	Non-clinical digital manometer	91.1 ± 26.4 (40.8–161.5)	
Maximal expiratory pressure (cmH_2_O)	Clinical digital manometer	113.8 ± 40.4 (54–244)	0.999 (0.998–0.999)
	Non-clinical digital manometer	114.5 ± 40.5 (54.3–243.8)	

SD: Standard deviation; ICC: Intraclass correlation coefficient; CI: confidence interval

**Fig 4:**
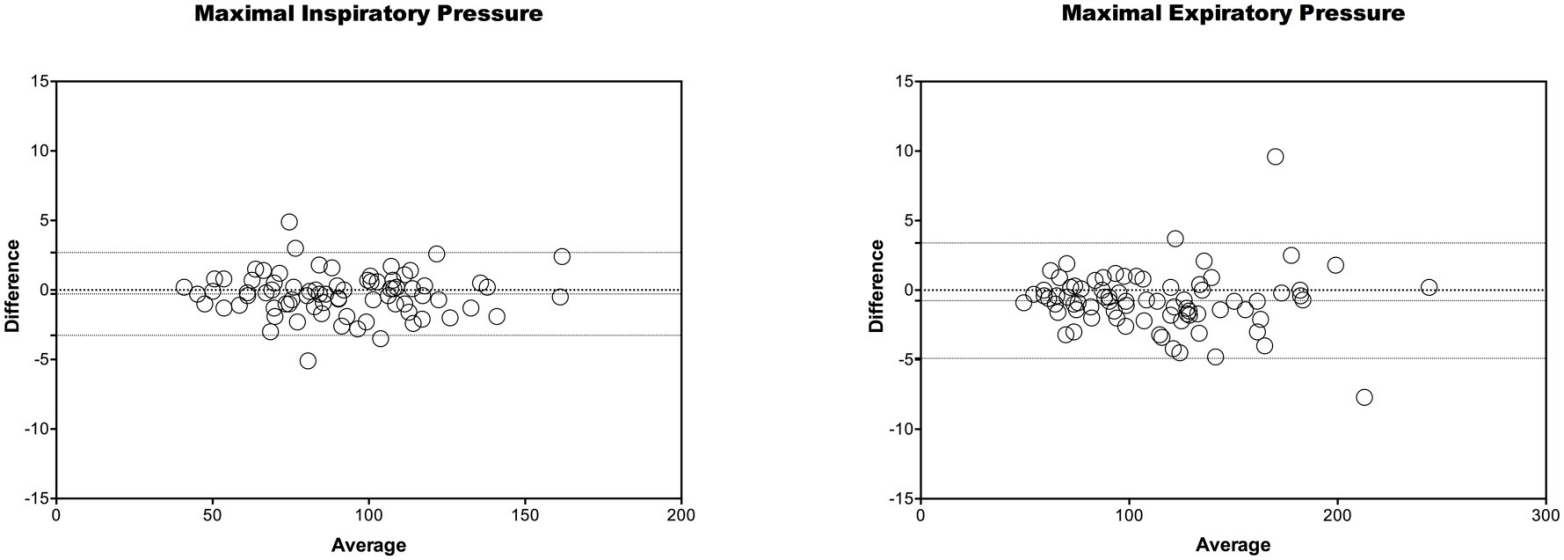
Bland-Altman plot show as the concordance of values of MIP and MEP. Each circle means the difference between the measured pressure of both manometer inline. Its method has a 95% of confidence limits, determinate for two standard deviations. If 95% of the sample is between lower and highest limits of confidence, exist agreement.

The ICC for MIP was 0.998 (IC 0.997–0.999) and for MEP was 0.999 (IC 0.998–0.999), which is classified as high concordance [15] (Table 2). In Fig 4, the Bland-Altman plot illustrates the level of agreement between the MIP and MEP values. The results of the Bland-Altman plot suggest low disagreement because the bias for the MIP was -0.29 cmH_2_O and for the MEP was 0.75 cmH_2_O.

## Discussion

Our results suggest that there is good agreement and concordance for values of maximal respiratory pressure obtained with clinical and non-clinical digital manometers in healthy participants. We obtained ICC of 0.998 and 0.999 for MIP and MEP, respectively which are above the 0.75 thresholds of good reliability [15]. Moreover, the Bland-Altman method confirmed that there was low disagreement for values of MIP and MEP.

The values obtained in our study were within the limits of normality recommended by clinical guidelines [1]. If we analyze the Bland Altman plot, we can see that there are very few data that escapes of the limits of agreement, Because the limits are narrow, we suggest that the two methods are essentially equivalent.

These results suggest that, from a technical point of view, the non-clinical digital manometer used in this protocol can measure maximum respiratory pressures with high reliability. However, in order for it to be used clinically, it must meet the requirements of health regulatory agencies in some countries. We verify that these devices are capable of measuring respiratory pressures, but the medical companies that manufacture or commercialize them must carry out safety studies so that they can be used on people.

We obtained parallel measurements to compare two devices, which differs from the methodology used in other studies. Previous studies have used the test-retest method or have compared two manometers used at different times [16–18]. We think that to compare pressure devices it is necessary to compare all devices in parallel, with the same dead space maneuver and lung volume, and therefore a similar pressure developed by the individual being tested.

In developing countries, such as those in South America, there is less availability of clinical digital manometers due principally to their expensive cost, which is not subsidized by public health programs (e.g., for programs of pulmonary rehabilitation) or high complex hospitals. In developing countries, most available manometers are analogs, which are difficult to calibrate and do not meet the criteria recommended by the ATS (i.e., digital devices with software for the analysis of collected data) [1]. Prolonged use of analog manometers can lead to measurement errors and reduced reliability. In contrast, digital manometers generally self-calibrate against ambient pressure, reducing the possibility of error. On the other hand, there is a wide availability of low-cost digital manometers, due to the massive use for the verification of industrial devices and processes. Our study was performed in healthy individuals, but in reality, this evaluation is used in patients with respiratory muscle weakness. However, similar studies [17–19] have also used healthy volunteers for validation of manometers because the reliable assessment of pressure must occur regardless of health status. For example, Maillard et al. [19] reported high reliability when using a maximal respiratory pressure manometer (Chest Scientific Instruments Ltd., United Kingdom) to measure MIP in healthy participants. Additionally, Maillard et al. assessed maximal respiratory pressures in a semirecumbent position, as they suggest participants should be seated during measurements, as in our protocol.

Validation studies of manometers have also been performed in participants with chronic respiratory disease, such as chronic obstructive pulmonary disease (COPD). Larson et al. [20] evaluated MIP across four consecutive weeks in 91 participants with COPD with an analog manometer (No 2000–200 cm Magnehelic pressure gauge, Dwyer Instruments, Michigan City, Ind.) that was calibrated and verified for a water column. The test-retest reliability coefficient was r = 0.97 for MIP measured at the third and fourth test.

Another important aspect that may contribute to differences between clinical and non-clinical digital manometers is the resolution of the instruments. While clinical devices have a resolution of 1 cmH_2_O, non-clinical devices have a resolution of 0.1 cmH_2_O. This difference likely has only a small effect on the agreement between measures, however, a device with greater precision (i.e., a non-clinical device) may hold an advantage in this respect.

Our study is subject to some limitations. First, we assessed young healthy individuals who do not represent the population of patients with respiratory muscle weakness. However, as previously stated, a suitable device should be able to accurately evaluate all individuals in all conditions. Second, the healthy individuals in this study had high-pressure values and we did not investigate people with lower pressure.

## Conclusion

Our results suggest that non-clinical digital manometers can accurately measure maximal respiratory pressure, as demonstrated in healthy individuals with a parallel measurement approach. Further validation at lower pressures and safety profiling among human subjects is needed.

## Supporting information

S1 Table Data of maximal inspiratory pressures and maximal expiratory pressures obtained in both devices. (XLSX)

## Abbreviations

BMI: Body mass index
COPD: Chronic obstructive pulmonary disease
FEV_1_: Forced expiratory volume at the first second
FVC: Forced vital capacity
ICC: Intraclass correlation coefficient
MEP: Maximal expiratory pressure
MIP: Maximal inspiratory pressure
TLC: Total lung capacity
